# First Report of *bla*_CTX–M–167_, *bla*_SHV–1_, and *bla*_TEM–1B_ Carrying *Klebsiella pneumonia* Showing High-Level Resistance to Carbapenems

**DOI:** 10.3389/fmicb.2022.916304

**Published:** 2022-07-07

**Authors:** Shirong Li, Siquan Shen, Li Ding, Renru Han, Yan Guo, Dandan Yin, Ming Guan, Fupin Hu

**Affiliations:** ^1^Deptartment of Laboratory Medicine, Huashan Hospital, Fudan University, Shanghai, China; ^2^Institute of Antibiotics, Huashan Hospital, Fudan University, Shanghai, China; ^3^Key Laboratory of Clinical Pharmacology of Antibiotics, Ministry of Health, Shanghai, China

**Keywords:** *Klebsiella pneumoniae*, *bla*_CTX–M–167_, *bla*_SHV–1_, *bla*_TEM–1B_, carbapenem

## Abstract

The prevalence of carbapenem-resistant *Klebsiella pneumoniae* is increasing. Although carbapenemase production is the main resistance mechanism of *K. pneumonia* to carbapenems, there are still some reports of non-carbapenemase-producing *K.pneumoniae* showing high-level resistance to carbapenems. In this study, we had also isolated a carbapenemase-negative carbapenem-resistant *K. pneumoniae* L204 from a patient with an asymptomatic urinary tract infection. Species identification was performed using MALDI-TOF MS, and carbapenemase-encoding genes were detected using both NG-test carba-5 and whole-genome sequencing. Antimicrobial susceptibility testing was performed by the broth microdilution method according to CLSI guidance. The results of antimicrobial susceptibility testing indicated that *K. pneumoniae* L204 was resistant to meropenem (MIC = 16 mg/L) and imipenem (MIC = 4 mg/L), but susceptible to ceftazidime-avibactam (MIC = 8 mg/L). Through whole-genome sequencing, several resistance genes had been identified, including *bla*_TEM–1B_, *bla*_CTX–M–167_, *bla*_SHV–1_, *aac(6’)-1b-cr*, *qnrS*, *aadA16*, *tet(A)*, *fosA*, *sul1*, and *mph(A)*. The efflux pump inhibition testing showed that the efflux pump was not involved in the resistance mechanism to carbapenems. The result of the conjugation experiment indicated that the plasmid with *bla*_CTX–M–167_ and *bla*_SHV–1_ was transferrable. The sodium dodecyl sulfate-polyacrylamide gel electrophoresis (SDS-PAGE) demonstrated that *K. pneumoniae* L204 only contained outer membrane porin OmpK35.

## Introduction

*Klebsiella pneumoniae* is one of the most common clinical opportunistic pathogens. It can be colonized in the respiratory and intestine tract of the human body (Chen et al., 2014; Hu et al., 2020), causing infection in the respiratory tract, urinary tract, and blood flow system (Amit et al., 2015; Lambelet et al., 2017). Immunosuppressive patients or those who use antibiotics for a long time are easy to be affected (Pantelidou et al., 2015; Xu et al., 2017; Arato et al., 2021). To control the infection caused by carbapenem-resistant *K. pneumoniae* (CRKP), a lot of new antimicrobial agents have appeared in recent years, bringing hope to patients with CRKP infection (Logan and Weinstein, 2017). Carbapenems have always been the first choice for clinicians to deal with carbapenem-sensitive *K. pneumoniae* infections due to their wide antibacterial spectrum and high stability (David et al., 2019). However, with the increase of CRKP, medical expenses and the mortality of patients are also increasing year by year (Shields et al., 2017), a systematic study on its resistance mechanism is helpful for clinicians to use antibiotics effectively. Although carbapenemase production is the main resistance mechanism of *K. pneumoniae* to carbapenems, numerous reports also have described non-carbapenemase-producing *K. pneumoniae* with high carbapenem levels. In this study, we found a common carbapenemase-negative (including *bla*_KPC_, *bla*_NDM_, *bla*_IMP_, *bla*_VIM_, and *bla*_OXA–48_) CRKP during routine antimicrobial susceptibility testing and carbapenemase testing (by NG-test Carba-5 kit). To clarify the resistance mechanism of CRKP to carbapenem including the existing of other carbapenemase genes or the new carbapenemase gene, we use various methods to study the mechanism that may mediate carbapenem resistance among this CRKP, and we reported a carbapenemase-negative CRKP clinical isolate bearing *bla*_CTX–M–167_ among *K. pneumoniae* coupled with the inactivation of OmpK36 from a patient. To our best knowledge, this is the first time to identify a *bla*_CTX–M–167_-positive *K. pneumoniae* strain in China.

## Materials and Methods

### Strains

A carbapenem-resistant *K. pneumoniae* L204 clinical strain was isolated from a urine sample of a patient with a urinary tract infection in Huashan Hospital, Fudan University in Shanghai, China. Species identification was performed using MALDI-TOF MS (bioMeìrieux, France). *Escherichia coli* ATCC25922 and *Pseudomonas aeruginosa* ATCC 27853 were used as controls for Antimicrobial Susceptibility Testing. *K. pneumoniae* ATCC 35657 that contains intact OmpK35 and OmpK36 used as a control for sodium dodecyl sulfate-polyacrylamide gel electrophoresis (SDS-PAGE).

### Antimicrobial Susceptibility Testing and Efflux Pump Inhibition Testing

The minimal inhibitory concentration (MIC) was determined by the broth microdilution method recommended by the Clinical and Laboratory Standards Institute (CLSI), and the results were interpreted according to CLSI M100-31th breakpoints for all agents except tigecycline, polymyxin, and cefepime–tazobactam (Yang et al., 2020; Clinical and Laboratory Standards Institute, 2021). Cefepime–tazobactam referred to the standards of cefepime in CLSI. Tigecycline MICs were interpreted using US FDA MIC breakpoints for *Enterobacterales* and polymyxin MICs were interpreted using the European Committee for Antimicrobial Susceptibility Testing (EUCAST) MIC breakpoints for *Enterobacterales*. Efflux pump inhibition testing (Sanchez-Carbonel et al., 2021) using Carbonyl cyanide 3-chlorophenylhydrazone (CCCP, 25 mg/L) as an inhibitor was conducted to determine whether the efflux pump takes a role in the resistance of *K. pneumoniae* L204 to imipenem and meropenem.

### Conjugation Experiment

A conjugation experiment was performed to explore the transferability of the plasmid. Briefly, the *bla*_CTX–M–167_ and *bla*_SHV–1_ -positive isolate *K. pneumoniae* L204 was used as the donor, while the *E. coli* J53 (azide resistant) was used as the recipient strain. Conjugants were selected on Mueller–Hinton (MH) agar supplemented with ampicillin (50 mg/L) and azide (100 mg/L). The presence of the *bla*_CTX–M–167_ and *bla*_SHV–1_ gene and other resistance genes in conjugants was confirmed by antimicrobial susceptibility, PCR, and DNA sequencing.

### Sodium Dodecyl Sulfate-Polyacrylamide Gel Electrophoresis

The cultured bacteria growing in 3ml LB broth was subjected to washing, crushed by ultrasonic, and then the membrane porin was obtained after centrifugation. Phosphate buffered saline (PBS) was used to resuspend the membrane protein. Extracted samples were separated using SDS-PAGE at a constant voltage of 130 V for about 50 min in both a 12% (w/v) polyacrylamide gel and a gradient gel (ExpressPlus™ PAGE Gel, GenScript, Piscataway, NJ, United States) as previous reported (Kurien and Scofield, 2012). The bands were detected using Coomassie brilliant blue R-250 staining.

### PCR and Whole Genome Sequencing Analysis

Five common carbapenemase genes (KPC, NDM, IMP, VIM, and OXA-48) were initially detected by PCR amplification and DNA sequencing (Gülmez et al., 2008; Poirel et al., 2011; Weiß et al., 2017). To fully understand the resistance genes among *K. pneumoniae* L204, in particular the carbapenemase, ESBL, and AmpC, we performed whole-genome sequencing analysis for this isolate. The genomic DNA of *K. pneumoniae* L204 was extracted by using a commercial Qiagen Midi kit (Qiagen, Hilden, Germany). The sequencing libraries were prepared using the Nextera XT DNA Library preparation protocol (Illumina, San Diego, CA, United States) and then the genomic DNA was subjected to Illumina (Illumina, San Diego, CA, United States) short-read sequencing (150 bp paired-end reads). Reads were trimmed with a sickle (GitHub) and were *de novo* assembled using SPAdes 3.12.0. Antimicrobial resistance genes analysis was performed using ResFinder 4.1^1^ and further verified using NCBI BLAST and the annotation process was done using RAST version 2.0.^2^

## Results

### Overview of the *bla*_CTX–M–167_ Producing Clinical Strain

*Klebsiella pneumoniae* L204 was isolated from a urine sample of a 55-year-old male patient with hydrocephalus who underwent a ventriculoperitoneal shunt. CT showed a small amount of inflammation in the lungs and a small amount of pleural effusion on both sides. A isolate of extended-spectrum β-lactamase-negative *K. pneumoniae* was initially isolated from the sputum, and cefoperazone-sulbactam (3g q8h) was given anti-infective treatment. Thereafter, the infection comes under control. On the 7th postoperative day, *K. pneumoniae* L204 was isolated from the urine and the anti-infection regimen was switched to meropenem (2g q8h) combined with amikacin (0.8g qd). The culture of the urine sample was negative three days later, and the patient was discharged 21 days after admission and was subsequently transferred to a rehabilitation hospital for further rehabilitation.

### Antimicrobial Susceptibility Testing and Efflux Pump Inhibition Testing

The antimicrobial susceptibility profiles of *K.pneumoniae* L204 to antimicrobial agents are presented in Table 1. The isolate was resistant to aztreonam (MIC ≥ 128 mg/L), ceftolozane-tazobactam (MIC ≥ 128 mg/L), meropenem (MIC = 16 mg/L), imipenem (MIC = 4 mg/L), levofloxacin (MIC ≥ 16mg/L), ciprofloxacin (MIC > 8mg/L), and tigecycline (MIC = 8 mg/L), but susceptible to amikacin (MIC = 2 mg/L), polymyxin B (MIC = 1 mg/L), and ceftazidime-avibactam (MIC = 8 mg/L). After adding CCCP, the MIC of meropenem and imipenem hasn’t changed indicating that the efflux pump may not play a role in the resistance mechanism of *K. pneumoniae* L204 to β-lactams such as ceftazidime, cefepime, piperacillin-tazobactam, aztreonam, meropenem, and imipenem.

**TABLE 1:** Susceptibility of *K. pneumoniae* L204 clinical isolate, conjugant, and recipient to antimicrobial agents.

Strains	β -Lactamase genes	MIC (mg/L)														
		CZA	IPM	MEM	CAZ	FEP	TZP	CZT	ATM	AMK	FPT	SXT	LEV	CIP	TGC	POL
*K. pneumoniae* L204	*bla*_TEM–1B_, *bla*_CTX–M–167_, *bla*_SHV–1_	8	4	16	>32	>128	>256	>128	>128	2	>64	>32	>16	>8	8	1
*K. pneumoniae* L204-CCCP (25 mg/L)	*bla*_TEM–1B_, *bla*_CTX–M–167_,*bla*_SHV–1_	8	4	16	>32	>128	>256	>128	>128	2	>64	>32	>16	>8	8	0.5
*E. coli* L204-C	*bla*_CTX–M–167_, *bla*_SHV–1_	0.5	0.25	≤0.03	2	32	4	0.5	16	4	≤0.03	>32	0.5	0.5	0.25	0.5
*E. coli* J53	–	0.5	0.25	≤0.03	0.5	≤0.06	4	0.5	≤1	≤1	≤0.03	≤0.25	0.125	≤0.06	0.125	0.25

*CZA, ceftazidime-avibactam; IPM, Imipenem; MEM, meropenem; CAZ, ceftazidime; FEP, cefepime; TZP, piperacillin-tazobactam; CZT, ceftolozane-tazobactam; ATM, aztreonam; AMK, amikacin; FPT, cefepime-tazobactam; SXT, trimethoprim-sulfamethoxazole; LEV, levofloxacin; CIP, ciprofloxacin; TGC, tigecycline; POL, polymyxin B.*

### Conjugation Experiment and SDS-PAGE

The *bla*_CTX–M–167_ and *bla*_SHV–1_-carrying plasmid was successfully transferred from *K. pneumoniae* L204 to *E. coli* J53, PCR-based sequencing demonstrated the presence of *bla*_CTX–M–167_ and *bla*_SHV–1_ in the conjugant *E. coli* L204-C. Compared with the recipient *E. coli* J53, the MICs of ceftazidime, cefepime, and aztreonam increased four times (from 0.5 to 2 mg/L), ≥512 times (from ≤0.06to 32 mg/L), and ≥16 times (from ≤1to 16 mg/L), respectively; while meropenem, imipenem, ceftazidime-avibactam, piperacillin-tazobactam, and cefepime-tazobactam make no difference for MIC between recipient and conjugant.

### Whole-Genome Sequencing Analysis and SDS-PAGE

**FIGURE 1:**
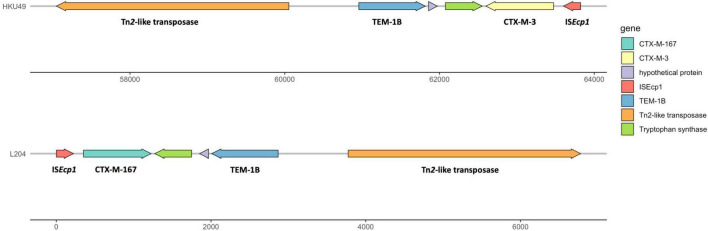
Multidrug resistance region (MRR) of *bla*_CTX–M–167_ in *K. pneumoniae*L204 and *bla*_CTX–M–3_ in HKU49. Resistance genes are indicated by yellow symbols. Transposon-related genes and insertion sequences are indicated by green symbols. Light gray shading indicated homologous regions (>99% DNA identity).

**FIGURE 2:**
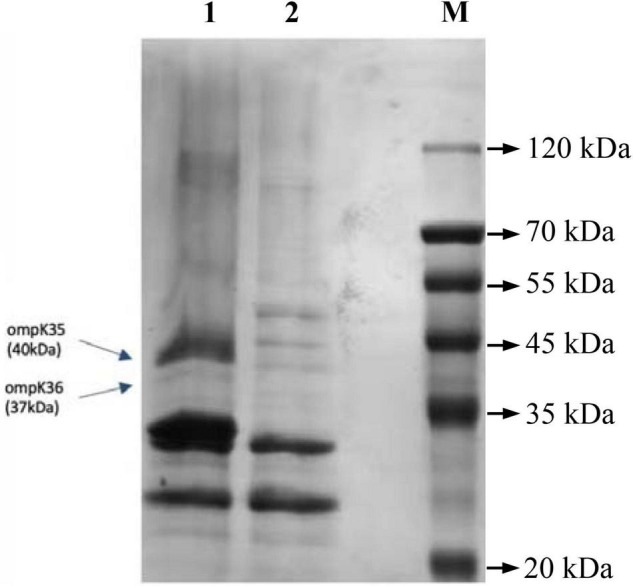
OMP analysis by SDS-PAGE. 1, *K. pneumoniae* ATCC35657; 2, *K. pneumoniae* L204; M, Marker.

The results of whole genome sequencing indicated that none of carbapenemase gene was detected in *K. pneumoniae* L204. However, a lot of resistance genes had been identified according to the whole genome sequencing, including the β-lactamase genes *bla*_TEM–1B_, *bla*_CTX–M–167_, and *bla*_SHV–1_, the fluoroquinolone resistance gene *qnrS1*, and the aminoglycoside resistance genes *aac(6’)-1b-cr* and aadA16, the tetracycline resistance gene *tet(A)*, the fosfomycin resistance gene *fosA*, the sulfonamide resistance gene *sul1*, and the macrolide resistance gene *mph(A).* Compared with *bla*_CTX–M–15_, *bla*_CTX–M–167_ has two amino acid substitutions: G at position 241 and 242 are replaced by C,D, respectively. Compared with *bla*_CTX–M–1_, except that the GG at 241-242 was replaced by CD, there was also a single base replacement, but it did not cause changes at the amino acid level. The genetic platform analysis revealed that the *bla*_CTX–M–67_ gene was flanked by ISEcp1 and Tn2-like transposase downstream and upstream, respectively. The full genetic environment surrounding *bla*_CTX–M–67_ is: Tn2-like transposase–*bla*_TEM–1B_–*bla*_CTX–M–67_–ISEcp1 (Figure 1). *K. pneumoniae* L204 has a complete ompk35, while Ompk36 has many mutations at many sites such as P.n218H and P.n217H were also reflected in whole gene sequencing. The result of multilocus sequence typing of *K. pneumonia* L204 indicated that the strain belonged to ST893. The results of SDS-PAGE of outer membrane protein showed that membrane porins Ompk35 (40 kDa) was complete while Ompk36 (37 kDa) in K. pneumonia L204 was not detected (Figure 2).

## Discussion

Extended-spectrum β-lactamase(ESBLs) are plasmid-mediated β-lactamases produced by gram-negative bacteria that are capable of conferring resistance to the penicillins, cephalosporins, and aztreonam by hydrolysis of these antibiotics, but usually have no activity against cephamycins or carbapenems (Paterson and Bonomo, 2005). According to the China Antimicrobial Surveillance Network, the detection rates of ESBL-producing strains in *E. coli*, *K. pneumoniae*, and *Proteus mirabilis* were 52.4, 41.5, and 38.2%, respectively, and *P. mirabilis* showed an increasing trend year by year.^3^ According to the Ambler classification, ESBLs are mostly from the class A TEM, SHV, and CTX-M families (Denkel et al., 2020; Verschuuren et al., 2021). CTX-M type ESBLs have surpassed TEM type and SHV type, and become the most widespread extended-spectrum β-lactamase in Gram-negative bacilli. Carrying CTX-M ESBLs with high hydrolysis activity to cephalosporin antibiotics is one of the main resistance mechanisms of *K. pneumonia* to antimicrobial agents, and their characteristics are carried by plasmids, which is also conducive to the generation, dissemination, and prevalence of new CTX-M ESBLs (Zhao and Hu, 2013). Although *Enterobacterales* from Asia, Europe, and America generally carry CTX-M ESBLs, and CTX-M-1 and CTX-M-9 are the main types, in different regions, the detection rate of the CTX-M is different (Daoud et al., 2015).

In this study, a multi-drug resistant *K. pneumoniae* L204 clinical strain with three β-Lactamase genes including *bla*_CTX–M–167_, *bla*_TEM–1B_, and *bla*_SHV–1_, only showed sensitivity to ceftazidime-avibactam, amikacin, and polymyxin B was isolated from a urine sample from a 55-year-old male patient with hydrocephalus who underwent a ventriculoperitoneal shunt. The result of the conjugation experiment showed that the plasmid containing *bla*_CTX–M–167_ and *bla*_SHV–1_ was successfully transferred from the donor to the recipient, and made the conjugant resistant to cefepime, and aztreonam while still susceptible to ceftazidime, meropenem, and imipenem.

To further explore the mechanism of resistance to carbapenems in *K. pneumonia* L204, we analyzed the efflux pump and outer membrane porins. The efflux pump inhibition testing indicated that the efflux pump does not play a role in the resistance of *K. pneumoniae* L204 to carbapenems. Combined with the previous study, Ompk36 loss may be one of the factors contributing to the resistance among ESBL-producing *K. pneumoniae*, and OmpK36 may play an essential role in the resistance or reduced susceptibility of *K. pneumoniae* to carbapenems coupled with ESBL and/or AmpC (Doménech-Sánchez et al., 2003; Tsai et al., 2011). In our study, The results of SDS-PAGE showed that OmpK36 was not detected in *K. pneumonia* L204, and whole-genome sequencing showed that it has many mutations at many sites such as P.n218H and P.n217H. we speculate that it was inactivated after genetic mutation and the loss of outer membrane porins may take the lead for the resistance of *K. pneumoniae* L204 to carbapenems, or other temporarily unknown mechanisms play an important role.

OmpK35 and OmpK36 belong to the Porin_1 (PF00267) group of bacterial outer membrane proteins. Both OmpK35 and OmpK36 form trimers composed of 16-stranded β-barrels integrated into the outer membrane, and the crystal structures of two of these proteins showed polar residues lining the internal porespolar molecules of less than 600 Da in size would permeate the channels formed by OmpK35 and OmpK36 (Dutzler et al., 1999; Acosta-Gutiérrez et al., 2018). Current knowledge on the regulation of porin gene expression in *K. pneumoniae* is limited, a study of *Klebsiella aerogenes* shows that overexpression of the small RNAs MicF and MicC can suppress the expression of omp35 and omp36, respectively (Hao et al., 2018).

Based on PLACNETw, the *bla*_CTX–M–167_ genes were located in contigs with homology to the plasmid that belonged to IncFII (K) type. The plasmid location of *bla*_CTX–M_ genes has already been reported, namely, compared with the chromosomal location of the resistance genes that can benefit the stable propagation of resistance, the plasmid location of the resistance genes can make the horizontal transmission easier (Zhang et al., 2017, 2022; Yang et al., 2021). BLAST comparison disclosed that the *bla*_CTX–M–167_ gene-environment of *K. pneumoniae* L204 shared >99% similarity with *bla*_CTX–M–3_-carrying plasmid pHKU49_CIP (GenBank accession number: MN543570) with 99% nucleotide identity and 100% query coverage, which was isolated in a *K.pneumonia* strain HKU49 from Hong Kong, China. The genetic platform analysis revealed that the *bla*_CTX–M_ gene was flanked by Tn2-like transposase and ISEcp1 downstream and upstream in both genetic environments, respectively. IS*Ecp1*, belonging to the IS*1380* family, is one of the most important insertion sequences associated with *bla*_CTX–M_ genes (Zhao and Hu, 2013; Leão et al., 2021). Tn3 transposon, which is conjugative, was also detected. These mobile genetic elements found in the plasmids may help promote excision and reintegration or transfer to other plasmids by horizontal propagation (Naseer and Sundsfjord, 2011; Cantón et al., 2012).

In summary, this is the first report of *K. pneumoniae* carrying *bla*_CTX–M–167_ globally, indicating a new evolutionary starting point for CTX-M ESBLs. Our study suggested that the monitoring and prevention of novel ESBLs should be strengthened to prevent the spread of multidrug-resistant gram-negative bacilli in healthcare facilities.

## Data Availability Statement

The datasets presented in this study can be found in online repositories. The names of the repository/repositories and accession number(s) can be found in the article/supplementary material.

## Ethics Statement

The study protocol was approved by the Institutional Review Board of Huashan Hospital, Fudan University (Number: 2018-408).

## Author Contributions

FH and MG designed the study. SL, SS, LD, RH, DY, and YG collected clinical samples and performed the experiments. SL, SS, FH, and MG analyzed the data. SS and SL wrote the manuscript. FH and MG reviewed the manuscript. All authors have read and agreed to the published version of the manuscript.

## Conflict of Interest

The authors declare that the research was conducted in the absence of any commercial or financial relationships that could be construed as a potential conflict of interest.

## Publisher’s Note

All claims expressed in this article are solely those of the authors and do not necessarily represent those of their affiliated organizations, or those of the publisher, the editors and the reviewers. Any product that may be evaluated in this article, or claim that may be made by its manufacturer, is not guaranteed or endorsed by the publisher.

## Footnotes

1
http://cge.cbs.dtu.dk/services/ResFinder/

2
https://rast.nmpdr.org

3
http://www.chinets.com/Data/GermYear
